# Development of animal models with chronic kidney disease-mineral and bone disorder based on clinical characteristics and pathogenesis

**DOI:** 10.3389/fendo.2025.1549562

**Published:** 2025-03-25

**Authors:** Biyu Tan, Weili Tang, Yan Zeng, Jian Liu, Xiaomei Du, Hongwei Su, Xianlun Pang, Lishang Liao, Qiongdan Hu

**Affiliations:** ^1^ Department of Nephrology, The Affiliated Traditional Chinese Medicine Hospital, Southwest Medical University, Sichuan, China; ^2^ Department of Orthopedics, The Affiliated Traditional Chinese Medicine Hospital of Southwest Medical University, Sichuan, China; ^3^ Department of Urology, The Affiliated Traditional Chinese Medicine Hospital, Southwest Medical University, Sichuan, China; ^4^ Department of Neurosurgery, The Affiliated Traditional Chinese Medicine Hospital, Southwest Medical University, Sichuan, China

**Keywords:** chronic kidney disease-mineral and bone disorder, renal osteodystrophy, animal models, clinical characteristics, pathogenesis

## Abstract

Chronic kidney disease–mineral and bone disorder (CKD-MBD) is a systemic complication of chronic kidney disease (CKD), resulting in high morbidity and mortality. However, effective treatment strategies are lacking. The pathogenesis of CKD-MBD is unclear but involves feedback mechanisms between calcium, phosphorus, parathyroid hormone, vitamin D and other factors, in addition to FGF23, Klotho, Wnt inhibitors, and activin A. Construction of a perfect animal model of CKD-MBD with clinical characteristics is important for in-depth study of disease development, pathological changes, targeted drug screening, and management of patients. Currently, the modeling methods of CKD-MBD include surgery, feeding and radiation. Additionally, the method of CKD-MBD modeling by surgical combined feeding is worth promoting because of short time, simplicity, and low mortality. Therefore, this review based on the pathogenesis and clinical features of CKD-MBD, combined with the current status of animal models, outlines the advantages and disadvantages of modeling methods, and provides a reference for further CKD-MBD research.

## 1 Introduction

Chronic kidney disease–mineral and bone disorder (CKD-MBD) significantly increases the incidence and mortality of fractures and cardiovascular diseases in patients, with high hospitalization rates and low quality of life (1–3), as well as incurring high medical costs and heavy social burdens. CKD-MBD accompanied by abnormal laboratory indicators, bone lesions, and calcification of blood vessels or other soft tissues has a prevalence of 33.3%–81% in developing countries (4, 5). The complicated pathogenesis of CKD-MBD involves fibroblast growth factor 23 (FGF23), α-Klotho (Klotho), Wnt inhibitors, activin A, and other factors. In addition to the general clinical manifestations of CKD disorders of calcium and phosphorus metabolism, secondary hyperparathyroidism (SHPT), persistent high levels of parathyroid hormone (PTH), abnormal vitamin D (VD) metabolism, bone abnormalities (manifested as bone turnover, mineralization, bone mass, linear bone growth, or bone strength abnormalities), and vascular or other soft tissue calcification are caused by CKD-MBD (6–9). Currently, the treatment of CKD-MBD is still complex, including dietary and lifestyle changes, adjustment of dialysis schedules, and the use of phosphate binders, VD, and calcimimetic agents (10). Management of patients with CKD-MBD faces higher demands because of considerations of therapeutic goals, adverse drug reactions, and health economics (11–14). Establishing a stable animal model of CKD-MBD based on its pathogenesis and clinical characteristics is crucial for the study of the disease, and provides an effective experimental tool for screening and testing of clinically effective drugs. Therefore, this review summarizes the current status of animal models of CKD-MBD and provides an overview of the pathogenesis, evaluation methods, modeling, and common problems of CKD-MBD.

## 2 Pathogenesis of CKD-MBD

**Figure 1:**
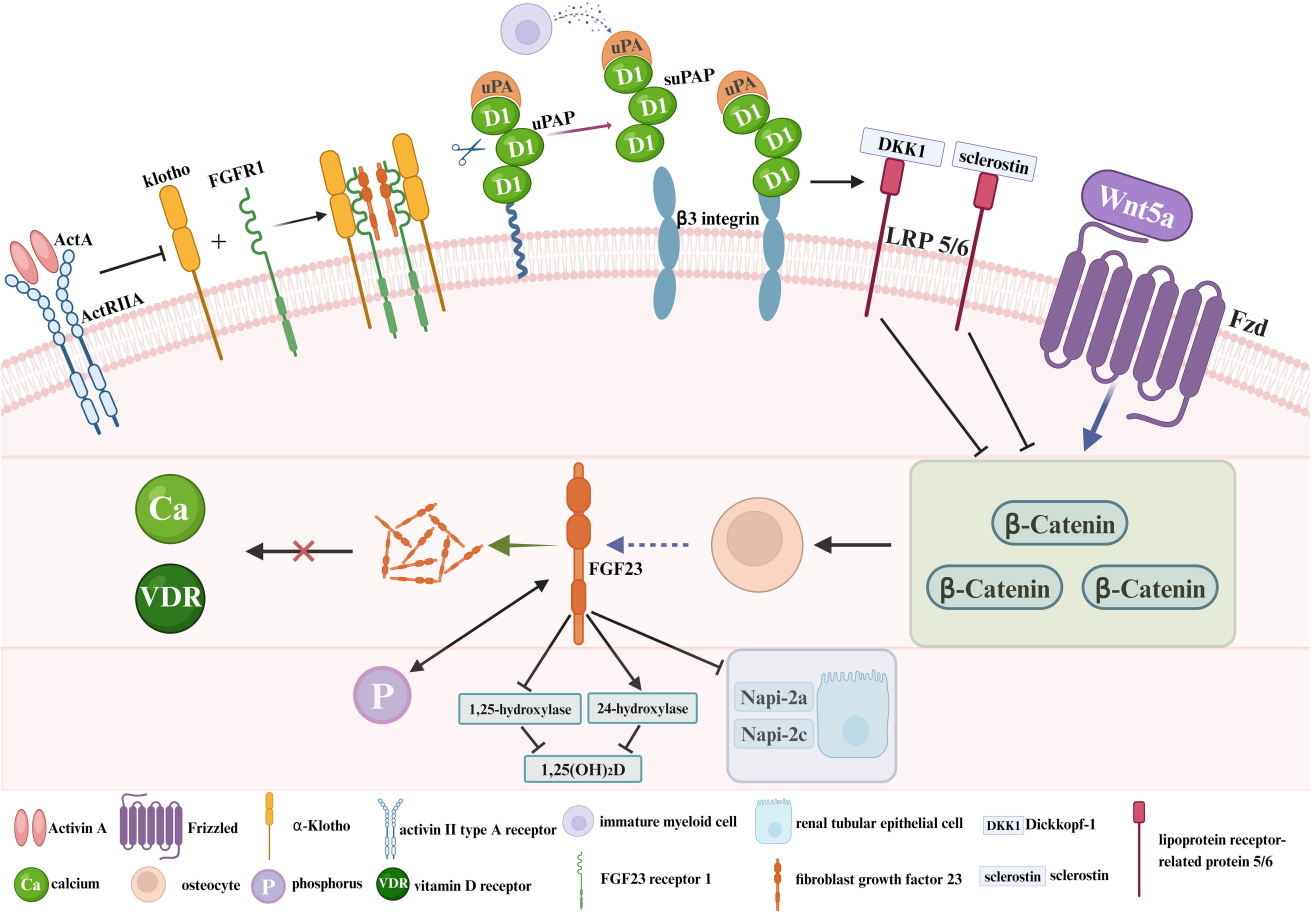
The pathogenesis of CKD-MBD. ActA, Activin A; ActRIIA, activin II type A receptor; Klotho, α-Klotho; FGFR1, FGF23 receptor 1; FGF23, fibroblast growth factor 23; uPA, urokinase plasminogen activator; uPAR, soluble urokinase receptor; suPAR, soluble urokinase plasminogen activator receptor; DKK1, Dickkopf-1; LRP 5/6, lipoprotein receptor-related protein 5/6; Fzd, Frizzled; Ca, calcium; VDR, vitamin D receptor; P, phosphorus; 1,25-(OH)_2_D, 1,25-dihydroxy-vitamin D.

The onset and progression of CKD-MBD involves feedback mechanisms between phosphate, calcium, PTH, VD, and other key factors (15, 16). FGF23, Klotho, Wnt inhibitors, activin A and circulating inflammatory biomarkers play different roles in the pathogenesis of CKD-MBD (**Figure 1**). FGF23 is derived from osteoblasts and plays an important role in VD and phosphate metabolism (17–19). By targeting proximal renal tubular epithelial cells, FGF23 decreases the surface expression of the sodium/phosphate cotransporter proteins NaPi-2a and NaPi-2c, thereby reducing renal phosphate reabsorption (20, 21). At the same time, FGF23 reduces intestinal phosphate absorption by down-regulating 1,25-hydroxylase activity and increasing 24-hydroxylase activity, thereby decreasing 1,25-dihydroxy-vitamin D (1,25-(OH)_2_D) synthesis (22–24). According to the early stages of CKD, the compensatory elevation of FGF23 levels can counteract hyperphosphatemia. Nevertheless, the prolonged FGF23 overdose reduces phosphate reabsorption by impairing the ability of the parathyroid gland to respond to calcium and vitamin D receptor (VDR) signaling pathway, thus exacerbating SHPT (25). Klotho is a calcium–phosphorus regulatory protein that has the ability to increase urinary phosphorus and prevent urinary calcium loss. It is tissue specific for FGF23, converting FGF23 receptor 1 (FGFR1) to a specific receptor for FGF23. In Klotho-deficient mice, vascular calcification, hyperphosphatemia due to abnormal calcium/phosphate metabolism, and shortened lifespan characterize the development (26, 27). The FGF23–Klotho axis can be disrupted in early CKD, which is characterized by decreased Klotho expression and increased FGF23 levels in serum. In the absence of Klotho, FGFR1 is underexpressed in the parathyroid glands and serum FGF23 levels are elevated, leading to a series of mineral metabolism disorders, SHPT, vascular calcification, and cardiac hypertrophy. Exogenous Klotho may ameliorate or prevent the progression of CKD-MBD (28). The Wnt signaling pathway (Wnt/beta-catenin) promotes bone formation and can affect bone remodeling by modulating the biological function of osteoblasts and osteoclasts. Wnt inhibitors (a combination of wingless and int) play a role in the pathogenesis of CKD-MBD. Wnt inhibitors, including Dickkopf-1 (Dkk1) and sclerostin, are secreted at increased levels in response to renal injury (29, 30). Overexpression of Dkk1 leads to decreased levels of beta-catenin, which reduces the number of osteoblasts, inhibits bone formation, and induces osteoclast differentiation and promotes bone resorption, leading to severe bone metabolic disorders (31). Activin A originates in renal-injured peritubular myofibroblasts and acts through the activin II type A receptor (ActRIIA) (32). In a mouse model, activation and inhibition of ActRIIA using the ligand trap RAP-011 (a fusion of the soluble extracellular structural domain of ActRIIA to a mouse IgG-Fc fragment) were separately evaluated for their roles in the pathogenesis of CKD-MBD (33). Activation of ActRIIA decreased Klotho expression and induced osteodystrophy and fibrosis, whereas inhibition of ActRIIA signaling was observed to reverse and ameliorate these changes (33, 34). Soluble urokinase receptor (uPAR) and soluble urokinase plasminogen activator receptor (suPAR), whose important cellular source is immature myeloid cells in the bone marrow (35), refer to circulating inflammatory biomarkers that play a pivotal role in the pathogenesis of renal diseases (36–39). As a cell membrane receptor distributed on the cell membranes of a wide range of immunoreactive cells and vascular endothelial cells, uPAR is involved in extracellular matrix degradation, inflammatory responses and tissue fibrosis by regulating the fibrinogen activation system (40). As the soluble form of uPAR shed in body fluids, suPAR, which is present in the peripheral blood circulation, is associated with inflammation and immune activation (41, 42). By virtue of impeding the formation of podocyte peduncles through activation of β3 integrin on glomerular podocyte membranes, suPAR is able to impair glomerular filtration and even cause pathological outcomes such as severe renal failure (36). However, there are currently no animal models that perfectly fit the clinical characteristics of CKD-MBD, due to the complex pathogenesis.

## 3 Modeling of CKD-MBD

Animal models were constructed by mimicking the development of human CKD-MBD. Most CKD-MBD animal models were formed by extending the modeling time of CKD animal models. However, the construction of animal models is required to be completed in a short period to facilitate experimental studies. Therefore, methods such as surgery, special diet, and radiation are sometimes adopted to accelerate the progression of CKD-MBD.

### 3.1 Surgical intervention

The surgical intervention method means that the kidney of the subject is removed or destroyed in some way, and varying degrees of kidney damage were caused, leading to CKD-MBD. 5/6 nephrectomy (Nx), unilateral ureteral obstruction (UUO), and electrocautery are used as common surgical modeling methods.

#### 3.1.1 5/6 Nx

**Figure 2:**
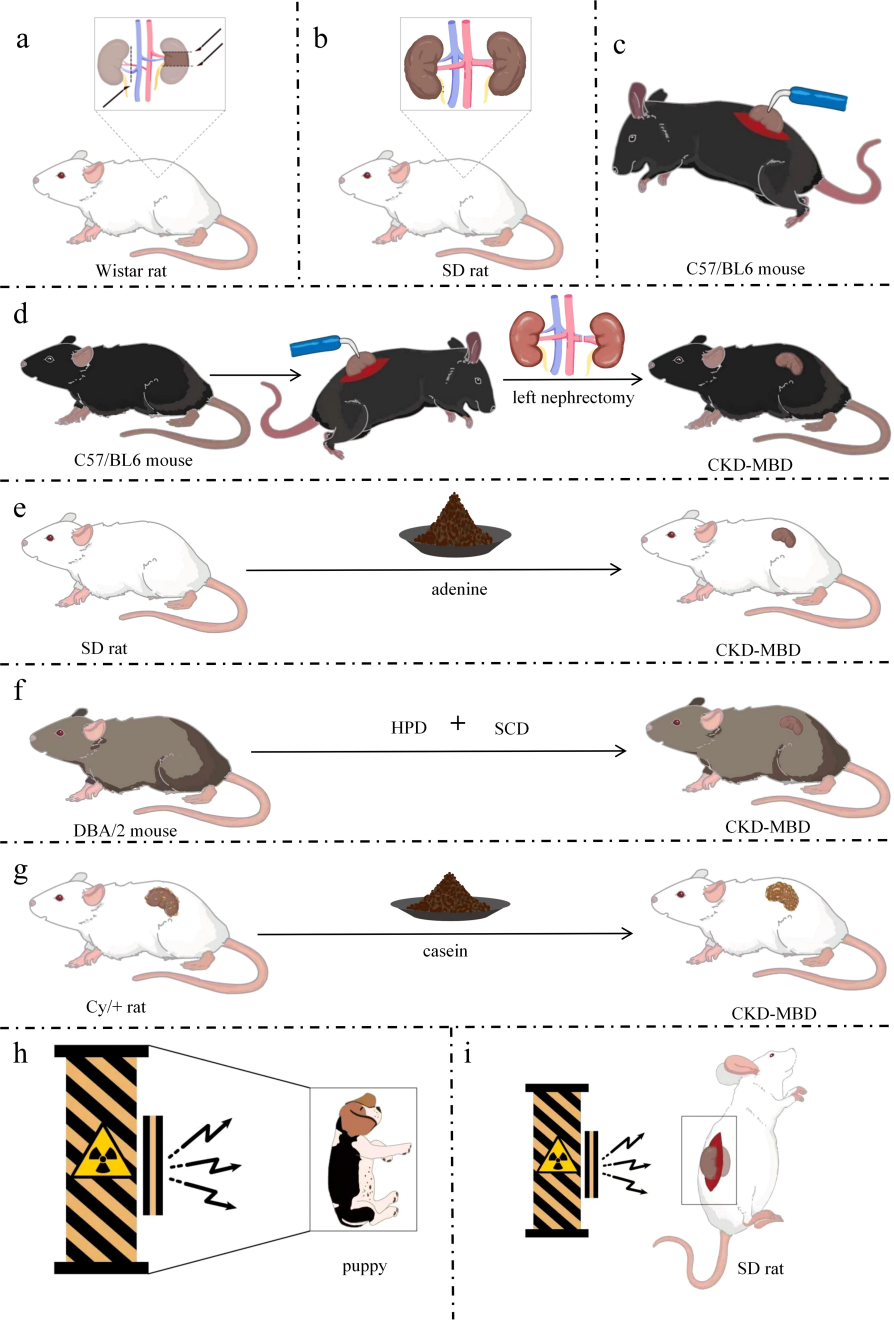
Simple models of CKD-MBD. **(a)** CKD-MBD model of 5/6 Nx; **(b)** CKD-MBD model of UUO; **(c)** CKD-MBD model of electrocautery; **(d)** CKD-MBD model of electrocautery combined with left nephrectomy; **(e)** CKD-MBD model of adenine alone diet; **(f)**CKD-MBD model of high-phosphorus diet; **(g)** CKD-MBD model of Cy/+ rat fed with casein diet; **(h)** CKD-MBD model of whole-body radiation in a puppy; **(i)** CKD-MBD model of localized radiation in rats. DBA/2, Dilute brown non-Agouti; Cy/+, a genetic model of polycystic kidney disease; SD, Sprague-Dawley; Nx, Nephrectomy; HPD, high-phosphate diet; SCD, standard chow diet; CKD-MBD, chronic kidney disease-mineral and bone disorder.

5/6 Nx is a widely used method. After removing 5/6 of rat kidneys, the residual renal units have the function of systemic blood filtration, thus leading to glomerular hyperfiltration, which further destroys glomerulosclerosis and the residual renal units, and results in the interstitial fibrotic lesions of chronic renal failure characterized mainly by glomerular hypertrophy and sclerosis (43, 44). Researchers have been preparing models of kidney diseases by 2/3 or 3/4 nephrectomy since 1889 when the first kidney-related animal models were created. However, no significant signs of proteinuria, hypertension, or myocardial hypertrophy were observed. Hence, Chauntin et al. (45) proposed the 5/6 Nx modeling method in 1932. This procedure was performed by first removing 2/3 of the kidney on one side of Wistar rats and then the entire kidney on the opposite side 1 week later. The rats were also characterized by significant proteinuria, nitrogen retention, hypertension, and cardiac hypertrophy after successful modeling. Jablonski et al. (46) reported that 150-day-old female Wistar rats were selected for a long-term renal osteodystrophy (ROD) model by the 5/6 Nx method in 1993 (**Figure 2a**). Blood samples were analyzed intermittently after surgery, and the rats were killed when 340 days old. Samples of the skull, residual kidney tissue, and bilateral femur, PTH, VD, alkaline phosphatase (ALP), calcium, and phosphorus were found to have varying degrees of change in the operated rats, and SHPT was observed, with a significant decrease in ALP indicating long-term obstruction of bone formation. The majority of the tested animals had reduced transverse cross-sectional area of the diaphysis and markedly increased bone resorption, with fibrous osteitis and long bone chondromalacia. In 2018, after constructing a CKD-MBD model by 5/6 Nx based on 8-week-old SD rats, the researchers found the elevated serum creatinine (Scr), phosphorus and intact parathyroid hormone (iPTH) levels and the decreased blood calcium levels compared with sham-operated rats (47, 48). At the same time, the model rats showed severe renal tubular injury and inflammatory interstitial cell infiltration. They also had extensive glomerulosclerosis, which was accompanied by a large number of dilated renal tubules and interstitial fibrosis. In addition, these CKD-MBD model rats displayed the significantly reduced bone mineral density. In 2021, founded on building a new CKD-MBD rat model using 5/6 Nx, Linna Liu et al. (49) revealed that serum urea nitrogen (BUN)and Cr levels were significantly elevated. At the 16th week of the experiment, the model rats showed significant lesions in the mesorectum of the aorta. Furthermore, the expression of bone morphogenetic protein 7 (BMP-7) was significantly down-regulated in the vertebrae of the CKD-MBD model rats, and the values of BMD, BV/TV, Tb, and Tb.Th were significantly decreased, and Tb.Sp was significantly increased. This model shows development of mixed bone impairments in uremic animals and can be used to study the early effects of CKD-MBD and the effects of different treatment options on bone. The subtotal nephrectomy animal model, a classic and mature model of CKD, has been mainly applied to the study of the pathological mechanisms of chronic renal failure. The model is equivalent to CKD stage 5 and GFR value is less than 15 ml/min/1.73m^2^. Researchers have used this model to evaluate and analyze the changes in bone tissue due to kidney injury as they have gained a better understanding of CKD. Typical bone abnormalities and vascular calcification are difficult to induce by 5/6-NX (50), owing to the high renal compensatory capacity of experimental rats, whose serum PTH, calcium, phosphorus, and ALP are not markedly altered. Therefore, more time is needed for developing the model of CKD-MBD. Infection and excessive blood loss are risks involved in this method of modeling, with greater surgical difficulty of control and high mortality (51, 52).

#### 3.1.2 UUO

CKD is a state of progressive renal fibrosis (53). The UUO model is well established and has been used to explore renal tubulointerstitial injury and progressive fibrosis (54). The hyperplasia of the renal tubular and interstitial cells and the aggregation and infiltration of macrophages and monocytes in the renal parenchyma induced by the UUO model ultimately lead to CKD-MBD due to tubulointerstitial fibrosis and tubular atrophy caused by the activation of the RAS system (55–57). For instance, 6-week-old male Sprague–Dawley (SD) rats (160–200 g) were anesthetized with isoflurane. The left nephron and ureter were exposed with a side abdominal incision, and the left ureter was ligated in two places with 3-0 sutures. After surgery, the rats were fed a high-phosphorus and low-calcium diet (1.2% Pi and 0.6% calcium) for 8 weeks (**Figure 2b**) (58). Histopathological analysis using micro-computed tomography (CT) and immunohistochemistry showed increased bone resorption in UUO model rats. However, the levels of Scr, phosphorus, intact PTH, and FGF23 were unremarkable in UUO model rats. The inconsistency in these results might stem from the compensatory renal excretory function of the contralateral kidney, rendering it difficult to ascertain the metabolic state of the bones solely based on serum biochemical markers. No further studies on vascular calcification in this model were performed at that time. The UUO model was dramatically disrupted by subtle biochemical changes and should be used with caution in studies of CKD-MBD. The UUO model is equivalent to CKD stage 4 and GFR in 15-29 ml/min/1.73m^2^. The UUO model is an ideal renal injury model to study the rapid progression of renal fibrosis with little effect on total glomerular filtration rate.

#### 3.1.3 Electrocautery combined with single nephrectomy

Electrocautery refers to the establishment of a model of CKD-MBD using needle-type electrocautery to damage the kidney cortex, causing inflammatory reactions and cortical fibrosis, resulting in elevated Scr and BUN. The renal failure model established by electrocoagulation of the bilateral renal cortex was first proposed by Gagnon et al. (59). Five-week-old C57/BL6 male mice were anesthetized with ether, whose kidneys were exposed through a 2-cm-long waist incision. The perirenal adipose tissue and capsule were then peeled off and the renal epidermis was subjected to electrocoagulation and electrocautery to a depth of ~1 mm, leaving the renal cortex 2 mm from the perirenal hilum unabraded. The same manipulations were performed on the left renal cortex 10 days later (**Figure 2c**). Scr and BUN levels were significantly increased 4 weeks after bilateral renal cortices were electrocoagulated. The model is equivalent to CKD stage 4 and GFR in 15-29 ml/min/1.73m^2^. The previous method of bilateral renal cortical electrocoagulation was modified by Lund et al. (60). The whole cortex of the right kidney was cauterized, excluding a 2-mm zone around the hilum, followed by total left nephrectomy 2 weeks later (**Figure 2d**). Elevated levels of BUN, serum phosphorus and PTH, hyperparathyroidism, conspicuous signs of femoral osteodystrophy (including reduced number and area of osteoblasts, lower rates of bone formation and bone mineralization deposition, decreased bone density, severe fibrosis around the trabeculae, and enlarged bone marrow cavities filled by fibrotic cells) were visualized after successful modeling. Renal electrocautery combined with single nephrectomy is a practical method for constructing CKD-MBD models. The model is equivalent to CKD stage 5 and GFR value is less than 15 ml/min/1.73m^2^. Restrictions of the electrocautery method are not just that complications can be caused at the beginning of the procedure, but also that the mortality of the subject is increased by complications of surgery, anesthesia, and late CKD (61).

### 3.2 Feeding intervention

Feeding modeling is a method by which animals are treated with various nephrotoxic drugs, foods, or special diets, causing renal unit injury, CKD, and then CKD-MBD. The causative agents include adenine, high-phosphate diet, casein diet, and doxorubicin.

#### 3.2.1 Adenine diet

By generating 2,8-dihydroxyadenine *in vivo* through the action of xanthine oxidase that is deposited in the glomerular and interstitial parts of the kidney, adenine helps form a foreign body granulomatous inflammation and block the lumen of the renal tubules to cause the corresponding cystic dilatation of the lumen of the renal tubules. As the disease progresses, a large number of lost renal units lead to CKD-MBD (62). Acute renal failure occurred in patients with Lesch–Nyhan syndrome treated with adenine in 1974 (63). An animal model of renal injury induced by adenine diet was first reported in 1986 (64). Male Wistar rats (~110 g) were fed particles containing 0.75% adenine (adenine dose: 270–320 mg/kg/day). Eight-week-old male SD rats (~200 g) were fed an adenine-containing diet (0.75% adenine) for 4 weeks (65), and the rats had raised levels of Scr, PTH, and phosphorus, reduced serum 1,25(OH)_2_D_3_, increased osteoid on the trabecular surface, active osteoblasts, and reduced cancellous bone mineral density (**Figure 2e**). In addition, 12-week-old male Wistar-Jcl rats were fed an adenine diet and exhibited a high turnover type of ROD (66). Male Wistar rats were fed a diet containing 0.25% adenine and low in vitamin K (67) to generate a CKD-MBD model. Chronic renal failure in rats can be induced by adenine diet, and also hyperparathyroidism and disorders of bone and calcium–phosphorus metabolism can be caused by this diet. Thus, a stable, highly reproducible, and highly transformative animal model of CKD-MBD was successfully established. More severe bone disease and vascular calcification can be manifested by the adenine-induced CKD-MBD animal model without invasive surgery (68, 69), and thus the difficulty of modeling and mortality are significantly reduced. However, some critical issues in elucidating the pathophysiological mechanisms of CKD-MBD exist in this model. Theoretically, bone metabolism is affected by systemic toxicity or organ-specific damage caused by adenine (62). The model is equivalent to CKD stage 5 and GFR value is less than 15 ml/min/1.73m^2^. Chronic renal failure is caused first, with renal bone disease then being caused by chronic renal failure as the first possible pathway. Bone metabolism directly affected by adenine is the second possible pathway, but the direct mechanisms by which it affects bone metabolism have not been reported. Weight loss, malnutrition, and systemic inflammation can be induced by an adenine diet.

#### 3.2.2 High-phosphorus diet

High-phosphorus diet increases the risk of decreased renal function (70) and has detrimental effects on bone health (71, 72). By increasing blood phosphorus levels, inhibiting calcium-sensitive receptor and vitamin D activation, and stimulating the overproduction of PTH and FGF23, high-phosphorus diets cause calcium and phosphorus metabolism disorders, enhanced bone resorption and mineralization disorders. Meanwhile, calcium phosphate deposition triggers ectopic calcification of the vasculature and soft tissues, ultimately leading to CKD-MBD (73). A novel model of CKD-MBD using only a high-phosphorus diet has been created in recent years (61). DBA/2 mice were fed with a high-phosphorus diet (20.2 g phosphorus, 9.4 g calcium,0.7 g magnesium, and 500 IU/kg vitamin D3) for 4 or 7 days, followed by standard chow diet (7.0 g phosphorus, 10.0 g calcium, 2.2 g magnesium, and 1000 IU/kg vitamin D3), and followed until day 84 (**Figure 2f**). The experimental mice were found to develop phosphate nephropathy, as demonstrated by tubular atrophy, interstitial fibrosis, reduced glomerular filtration rate, elevated serum urea, as well as SHPT, arterial calcification, and reduced tibial bone volume and mineralization. The model is equivalent to CKD stage 5 and GFR value is less than 15 ml/min/1.73m^2^. The high mortality in animals due to surgical modeling was reduced because the model excluded the effects of surgical intervention, and because the low turnover bone disease was described for the first time. In addition, progression of CKD-MBD was better simulated by this model, rendering it a new, noninvasive, easy-to-perform, and reproducible model. However, limitations of the model include prolonged breeding and close monitoring of animals. The mortality of the mice fed high-phosphorus diet for >10 days increases rapidly. Lastly, researchers found that the model differed in susceptibility to vascular calcification by comparing CKD patients to this CKD mouse model (61).

#### 3.2.3 Casein diet

Casein diet by regulating phosphorus and calcium intake result in hyperphosphatemia and increased PTH secretion, accompanied by renal fibrosis and mineral metabolism disorders, which ultimately leads to CKD-MBD (74, 75). The heterozygous Cy/+ rat is a genetic model of polycystic kidney disease that can be developed into CKD-MBD on a special diet (75). Male Cy/+ rats are consistent in the progressive development of nephropathy and have several features of advanced CKD-MBD (76). Cy/+ rats were fed with a high-casein diet (18% casein-based protein, 0.7% phosphate, 0.7% calcium, and 5% fat) and were sampled at 10, 34, and 38 weeks of age, which showed persistent azotemia beginning at 10 weeks of age, hyperphosphatemia, and hyperparathyroidism at 34 weeks of age, vascular calcification at 38 weeks of age, and uremia at ~40 weeks of age (**Figure 2g**). Dietary protein type affecting the progression of CKD-MBD and renal dysfunction were confirmed by this model, and casein was introduced as a novel dietary modeling method, alongside further exploration of the mechanisms involved; namely that a casein-based diet increases the concentration of FGF23, resulting in hyperphosphatemia to complete the modeling. The model is equivalent to CKD stage 5 and GFR value is less than 15 ml/min/1.73m^2^. This model, as the first CKD-MBD model that occurs spontaneously under a normal phosphorus diet without surgical or pharmacological involvement, can simulate the development of human CKD, study the early changes of CKD-MBD, and evaluate the effects of different dietary regimens on the course of CKD-MBD.

### 3.3 Radiation

Radiation is an animal modeling method to induce CKD-MBD, similar to bone disease due to chronic renal failure. The systemic radiation contributes to anemia and immunosuppression by destroying the hematopoietic function of the bone marrow (77). At the same time, systemic radiation induces oxidative stress and inflammation (78), which exacerbates mineral metabolism disorders, ultimately leading to CKD-MBD. The dose and timing of radiation are important because too small a dose makes it difficult to produce visible kidney damage, while too large a dose can cause gastrointestinal damage (79, 80). Two-day-old puppies were exposed to sublethal doses of 60Co gamma radiation in 1981 (**Figure 2h**) (81), inducing varying degrees of renal failure, with hyperparathyroidism, altered osteochondrosis, increased bone remodeling, and reduced bone mineral density. The model is equivalent to CKD stage 5 and GFR value is less than 15 ml/min/1.73m^2^. Therefore, the radiation method can be applied to the study of CKD-MBD in humans. The local radiation induces renal failure by directly damaging renal tissues, which in turn leads to CKD-MBD. The direct damage to renal tissues can cause renal hypoplasia, which in turn triggers inflammatory reactions and fibrosis, as well as disorders of mineral metabolism, ultimately leading to the pathologic features of CKD-MBD (82).

The method of inducing impaired bone metabolism by establishing renal injury through localized radiation to the kidneys based on whole-body radiation was proposed by Ming-Yu Wu et al. (82). The bilateral kidneys of 3-month-old male SD rats (280–310 g) were exposed through longitudinal incisions on both sides of the spine. The rats were fixed in the lateral recumbent position on a radiation rat mold, and the tissues other than the kidneys were shielded by lead plates. The exposed kidneys were irradiated by gamma rays at 15 Gy (**Figure 2i**). Indicators of bone mass, three-point bending load on the femur, and compressive load on the lumbar spine were significantly reduced in the subjects after 3 months of local kidney irradiation. Bone morphology tests showed diluted bone trabeculae and accelerated bone conversion. In addition, renal-radiation-injury-induced bone metabolism disorders are similar in clinical manifestations to renal bone disease due to chronic renal failure, with frequent manifestations of osteoporosis, fibrous osteitis, resting bone disease, and osteochondrosis, which predispose to fractures. The model is equivalent to CKD stage 5 and GFR value is less than 15 ml/min/1.73m^2^. In conclusion, definite bone changes can be caused by systemic radiation and the modeling process is similar to the progression of renal failure. However, clinical features more similar to CKD-MBD are demonstrated by localized radiation of the kidneys. Shortcomings of the radiation method are the long modeling times due to slowly developing radiation damage to the kidneys. Different nephrotoxicity thresholds are available in different radiological entities (83), so the difficulty of modeling is heightened by the radiation dose and length of time required for radiation.

### 3.4 Improved models

The improved versions of the models refer to the derivation or combination of the above models to obtain a model with a shorter modeling time, greater efficiency, and greater suitability for research purposes.

#### 3.4.1 Partial nephrectomy combined with high-phosphorus diet

**Figure 3:**
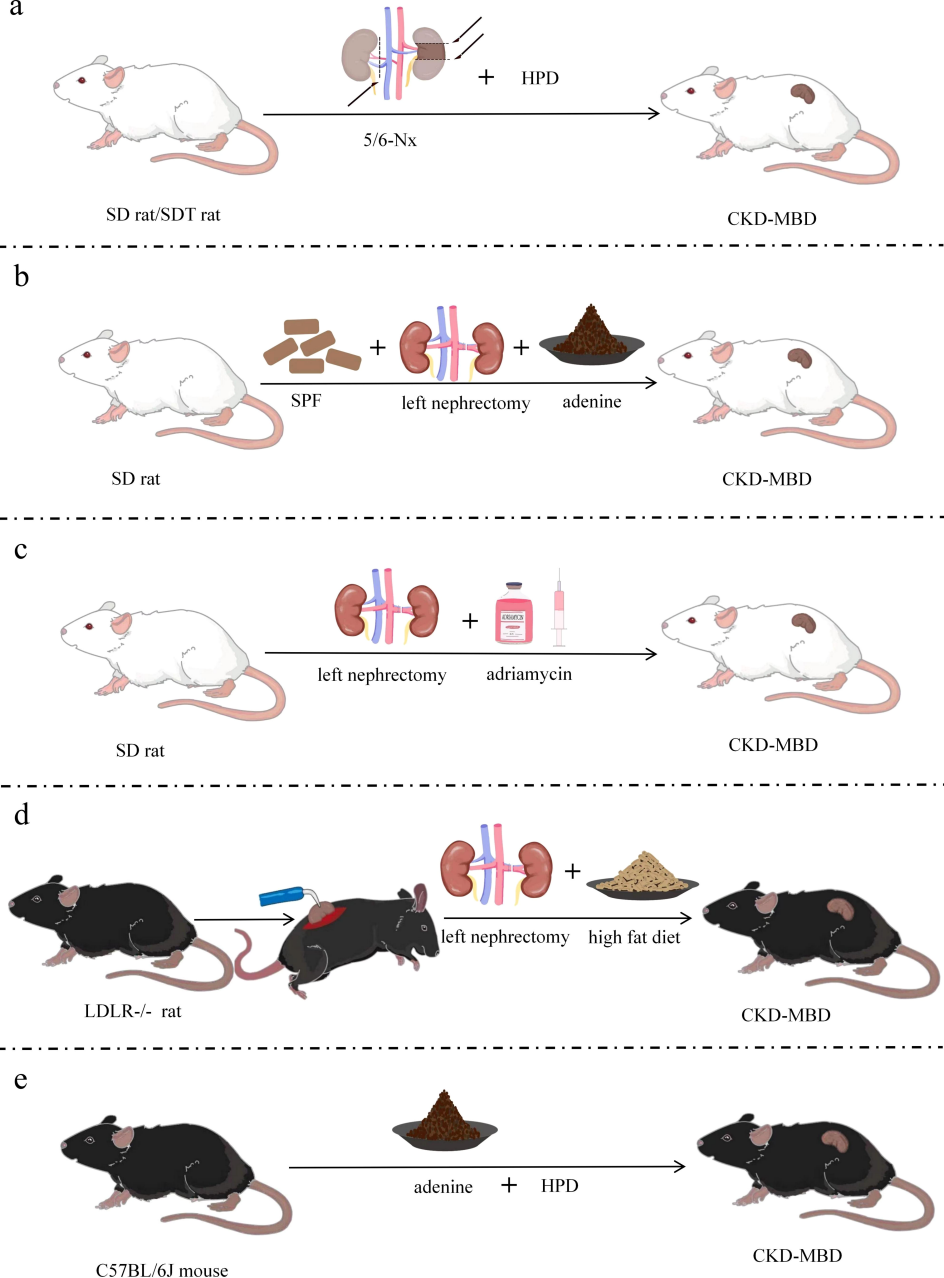
Improved models of CKD-MBD. **(a)** CKD-MBD model of partial nephrectomy combined with high-phosphorus diet; **(b)** CKD-MBD model of unilateral nephrectomy combined with adenine diet; **(c)** CKD-MBD model of nephrectomy combined with doxorubicin; **(d)** CKD-MBD model of LDLR^-^/^-^ mice with electrocautery combined with nephrectomy and special dietary intervention; **(e)** CKD-MBD model of fed with high-phosphorus and adenine diet. SD, Sprague-Dawley; SDT, spontaneously diabetic Torii; LDLR=low density lipoprotein receptor; Nx, Nephrectomy; HPD, high-phosphate diet; SCD, standard chow diet; SPF, specific pathogen-free; CKD-MBD, chronic kidney disease-mineral and bone disorder.

5/6 NX brings severe damage to renal function, such as increasing the burden on the residual kidneys and decreasing the glomerular filtration rate to a high degree. Founded on this, a high-phosphorus diet further exacerbates the abnormalities of bone metabolism and vascular calcification, thus leading to the development of CKD-MBD (84). Modeling with a high-phosphorus diet based on a 5/6 Nx model can achieve better modeling results for CKD-MBD in a short period. Male Wistar rats (200–225 g) received a high-phosphorus diet (0.8% calcium and 0.93% phosphorus) for 3 weeks before starting two-stage 5/6 Nx (85). Two branches of the left renal artery were ligated under anesthesia, followed by removal of the right kidney 2 weeks later (**Figure 3a**). Execution and sampling were performed at 6 and 12 weeks, followed by testing that revealed elevated PTH, hyperphosphatemia, hypocalcemia, and SHPT, as well as increased mineral deposition rates, bone formation rates, osteoblast perimeter, and erosion perimeter. The model can be used to assess the effects of dietary phosphorus and the severity of renal failure on morphological changes in bone histology and various biochemical markers. Similarly, Scr, FGF23, and phosphorus were significantly elevated, and total serum ALP activity was increased with severe SHPT in 5/6 Nx mice fed a high-phosphorus diet (2.0% calcium, 1.25% phosphorus, 20% lactose, and 600 IU VD/kg) for 8 weeks postoperatively (86). Low total and cortical bone density of the spine and proximal tibial epiphysis, as well as significant signs of impaired bone mineralization were detected. The CKD-MBD rat model was developed by Linna Liu et al. (49) using 5/6 Nx combined with a high-phosphorus diet (0.5 g sodium dihydrogen phosphate, 0.5 g sodium dihydrogen phosphate). Alternatively, male SDT and SD rats were used by Kentaro Watanabe et al. and were divided into experimental and control groups (48). The experimental group had 2/3 of their left kidney removed at 8 weeks of age, followed by CKD being established by total nephrectomy of the right kidney 1 week later. Then CKD-MBD animal model was established by feeding a high-phosphorus diet (1.0% calcium and 1.2% phosphate) at 10 weeks of age. SDT-Nx rats that had undergone 5/6 Nx compared with SD nephrectomy rats by 20 weeks of age showed more dramatic changes in CKD-MBD parameters, including vascular calcification, serum PTH, FGF23, serum calcium and phosphorus levels, and urinary excretion of calcium and phosphorus. Partial nephrectomy combined with a high-phosphorus diet is regarded as an optimal mouse model of chronic renal failure, with characteristics such as malnutrition, hypertension, and disturbed calcium and phosphorus metabolism, thus making an ideal model for studying CKD-MBD. The model is equivalent to CKD stage 5 and GFR value is less than 15 ml/min/1.73m^2^. SDT-Nx rats can also be used to examine the pathophysiology of CKD-MBD.

#### 3.4.2 Unilateral nephrectomy combined with adenine diet

Left nephrectomy aggravates the burden on the remaining kidneys, resulting in reduced renal function. Moreover, because adenine is nephrotoxic, an adenine diet causes interstitial fibrosis and tubular damage, which further impairs residual renal function and triggers abnormal mineral metabolism, ultimately leading to CKD-MBD (51). The experiment was conducted using 200–220 g Male SD rats (87) housed at 22 ± 3°C with 50± 0% humidity on a 12-h light/dark cycle, and were fed standard rat chow of specific pathogen-free grade. The rats were subjected to left-sided nephrectomy on day 7 and given 2% adenine (150 mg/kg/day) on days 8–21 (**Figure 3b**). Renal insufficiency, tubular interstitial injury, disturbance of calcium and phosphorus metabolism, and bone abnormalities were found 3 weeks after the induction of renal injury. The model is equivalent to CKD stage 5 and GFR value is less than 15 ml/min/1.73m^2^. Significant vascular calcification was present in this modified CKD-MBD rat model due to the use of adenine, and renal injury and bone abnormalities were more easily studied.

#### 3.4.3 Nephrectomy combined with Adriamycin

The compensatory hyperfiltration state of the residual kidneys after left nephrectomy results in intraglomerular high pressure, proteinuria and oxidative stress, which further harms renal structure. Additionally, adriamycin causes glomerular shrinkage, glomerulosclerosis, tubular atrophy and tubulointerstitial fibrosis (88), and left nephrectomy combined with adriamycin leads to deterioration of renal function and the development of bone pathology, ultimately leading to CKD-MBD. Nephrotoxic drugs were used by Liu et al. (89) based on 1/2 nephrectomy; i.e., the surgical modeling method was combined with the drug–food modeling method. Thus, a new, short-term model of CKD-MBD was formed. Male SD rats of 120–150 g were selected and anesthetized under pentobarbital (60 mg/kg) for left nephrectomy with intravenous doxorubicin (dissolved in 0.9% saline, 5 mg/kg), whereby an ROD model was created, and experimental sampling was performed at various times after surgery (**Figure 3c**). Marked increases in BUN, Scr, Uric Acid(UA), and Urea-Creatinine Ratio (UCR), and decreases in serum albumin were indicated in subjects with late ROD (1 week < duration of disease ≤ 1 month). Overt renal injury was also found. Low transforming bone lesions were revealed by bone morphology that were characterized by marked decreases in bone formation rate, osteoclasts, osteoblasts, and trabecular volume thickness, as well as a significant increase in osteoid volume. This experimental model is proposed as a highly reproducible model of kidney injury, whose time to induction is short and time to injury is predictable and consistent. Therefore, this model can also be used to test interventions that exacerbate or prevent kidney injury. The model is equivalent to CKD stage 4 and GFR in 15-29 ml/min/1.73m^2^. Additionally, the process of human CKD development can be better simulated on account of the high similarity between the type of structural and functional impairment of the model and that of human chronic proteinuria nephropathy.

#### 3.4.4 Electrocautery combined with nephrectomy and special dietary intervention in LDLR^-^/^-^ mice

By directly injuring the renal tissues, both left nephrectomy and electrocautery trigger inflammatory reactions and fibrosis, causing hyperphosphatemia, increased PTH secretion and abnormal bone metabolism, ultimately leading to CKD-MBD (50). Therefore, better modeling results will be achieved by combining the two procedures. LDLR^-^/^-^ mice were used by Davies in the preparation of this model (90), with standard diet-fed mice being given a high-fat diet for 2 weeks at 10 weeks of age. The experimental manipulations were carried out following the procedure previously described by Gagnon (59) and Lund (60) at 12 weeks of age. The rate of mineral deposition in the cancellous bone of the distal femur was significantly reduced, osteoblasts were reduced, bone formation was slowed, and low turnover of osteodystrophy was observed in mice fed a high-fat diet. The right kidney of 12-week-old LDLR^-^/^-^ mice was subjected to electrocautery through a 2-cm lateral incision pair (91), with mild and moderate kidney injury being produced according to the degree of cautery. The left kidney was removed through a similar incision in mice at 14 weeks, followed feeding a high-fat diet until 22 or 28 weeks (**Figure 3d**). The final experiment showed suppressed bone formation rate, decreased cortical bone density, decreased bone area, increased osteoclast secretion, and vascular calcification. In addition, BUN, calcium, phosphate, and PTH were elevated at week 28. The degree of renal injury was lowered by reducing the area of electrocautery in the right kidney. Early CKD (stages 2 and 3) in the model group was judged by utilizing inulin clearance, and CKD-MBD appeared early. The model is equivalent to CKD stage 5 and GFR value is less than 15 ml/min/1.73m^2^.

#### 3.4.5 Combined high-phosphorus and adenine diet

Adenine-fed renal pathology stems from the formation of 2,8-dihydroxyadenine, an adenine metabolite that crystallizes in renal tubules, which leads to an inflammatory response, oxidative stress, tubular atrophy and renal parenchymal fibrosis. A high-phosphorus diet increases the phosphorus load in the body and damaged kidneys can’t excrete phosphorus efficiently, leading to elevated blood phosphorus and stimulating increased secretion of PTH, which triggers disorders of mineral metabolism and ultimately leads to CKD-MBD. Eight-week-old male C57BL/6J mice were used by Takashi et al. in 2017 (73). A novel CKD mouse model with adenine and high-phosphate diet for assessing the progression of hyperphosphate and associated mineral bone disease, and the longer the high-phosphorus diet was fed, the greater the volume of calcification was found. Twenty-week-old male C57/BL6J mice were recently used to create a CKD-MBD model (92) in which CKD was induced by feeding a 0.2% adenine and 0.8% phosphorus diet for 6 weeks, followed by induction of CKD-MBD by feeding a 0.2% adenine and 1.8% phosphorus diet for 6 weeks (**Figure 3e**). Elevated creatinine and phosphorus, decreased calcium, SHPT, thin and irregular femoral cortex, visibly reduced cortical bone mineral density and cortical bone thickness, and reduction in bone volume and trabecular number were detected after successful modeling. The model is equivalent to CKD stage 5 and GFR value is less than 15 ml/min/1.73m^2^. The complications associated with medial arterial calcification and ROD in patients with CKD are mimicked by this modeling approach, and developed severe vascular calcification without surgery.

## 4 Quality evaluation of animal models of CKD-MBD

No clear assessment criteria are seen in animal models with CKD-MBD, and serum biochemical tests, renal histopathological tests, bone tissue-related index tests, and vascular calcification are commonly used for the evaluation and diagnosis of CKD-MBD (93, 94).

### 4.1 Serum biochemical tests

CKD-MBD is treated as a disease secondary to CKD. CKD-MBD is regarded as a secondary disease of chronic kidney disease (CKD), and its animal model needs to be evaluated by serum biochemical tests to reflect the key indicators of renal function and mineral metabolism disorders. The commonly used assays include serum Scr, BUN, calcium, phosphorus, 1,25-(OH)_2_D, PTH (95, 96) and ALP (73, 97, 98). Additionally, in order to improve the validity and reproducibility of the model, it is recommended to incorporate statistical analyses to validate the sensitivity and specificity of these assays, and to ensure the consistency of the experimental conditions (e.g., feed formulations, surgical procedures) in order to minimize bias due to individual differences.

### 4.2 Renal histopathology

Renal histopathology serves as an important tool for evaluating animal models of CKD-MBD. Pathological changes in the kidneys, including glomeruli, tubules, and interstitium, are usually visualized under light microscopy after hematoxylin–eosin staining, peroxynitrite Schiff staining, and Masson staining. To enhance the clinical relevance of the model, quantitative analyses (e.g., glomerular sclerosis rate, percentage of fibrotic area) should be combined to quantify the pathological changes and compared with the pathological characteristics of human CKD-MBD patients to validate the representativeness of the model.

### 4.3 Bone tissue-related indicators

Bone tissue-related index tests have been used as an important indicator for the diagnosis of CKD-MBD. The lumbar spine, femur, and tibia are often tested in experiments, with the femur being the most commonly tested (75, 87).The presence of abnormalities in bone transformation, mineralization, bone volume, linear bone growth, or bone strength is clarified by detecting bone mineral density (99), hematoxylin–eosin staining of bone sections (87, 100), Masson staining, tartrate-resistant acid phosphatase (TRAP staining), Goldner’s staining (101), immunohistochemical staining, and micro-CT, and the type of bone transformation can be predicted by the results of the assay (102–104). By combination with the dynamic bone metabolism markers detection, the progression pattern of bone lesions can be observed through long-term follow-up, thus enhancing the validity, time-dependence and clinical relevance of the model.

### 4.4 Vascular calcification

Damage to the cardiovascular system in CKD-MBD is considered an important factor in mortality (1). Experimental models are often assessed by vascular calcium content measurement, Von Kossa staining of aortic segments, and percentage of aortic calcified plaque area (50, 105, 106). To improve the reproducibility and clinical relevance of the model, it can be combined with vascular endothelial function test as well as inflammatory factor test to comprehensively reflect the pathological mechanism of vascular calcification and to be compared with the vascular lesion characteristics of human CKD-MBD patients.

### 4.5 Other assessment methods

The establishment of CKD-MBD animal models should also consider the validity, reproducibility and clinical relevance. Firstly, in terms of the model validity, it can be statistically verified that the key features of the model (e.g., serum biochemical indexes, pathological changes and vascular calcification) are at the expected level and ensure that the experimental results are significant and biologically meaningful. Secondly, in terms of the model reproducibility, a standardized operation procedure, encompassing experimental design, animal selection, surgical operation and detection methods, can be established to verify the stability and consistency of the model through multiple batches of experiments. Finally, the clinical relevance of the model can be compared with the clinical data and pathological features of human CKD-MBD patients to verify whether the animal model can accurately simulate the pathophysiological process of human disease.

A well-established model of CKD-MBD should resemble the alterations in bone, renal function, and electrolytes of clinical patients, in addition to the above-mentioned indicators being examined. Also, the model should be altered by chronic renal failure and not by other diseases or drugs. The model is required to be broadly representative, highly stable, and reproducible, while simple to operate, take a short time to achieve, and be capable of representing renal bone disease from various causes.

## 5 Discussion and conclusion

The clinical presentation of patients with CKD-MBD varies with the main metabolic abnormalities and characteristic bone disease of the patient. CKD-MBD is primarily characterized by ROD, leading to weakness, fractures, bone and muscle pain, and ischemic necrosis. Hyperconversion osteodystrophy, hypoconversion osteodystrophy, mixed ROD, and β2-microglobulin amyloidosis osteodystrophy are specifically included in ROD. The treatment of CKD-MBD has so far focused on phosphate retention, abnormal VD metabolism, and PTH disruption, but the strategies have largely proved to be unsuccessful. Recently, a single modeling method has been found to have a long modeling time and other obvious shortcomings as the animal models of CKD-MBD have been improved. For example, the mortality rate of 5/6 Nx is high, the experimental subjects of high-phosphorus diet modeling are limited and have different sensitivities, and the radiation dose and duration of radiation modeling are difficult to control. The modified modeling methods were found to be noticeably more advantageous than single modeling methods concerning the modeling time. Unilateral nephrectomy combined with adenine diet is recommended considering the cost of modeling and the difficulty of the operation. Previous surgical modeling methods have resulted in high mortality rates due to postoperative infections and other factors. However, penicillin given to SD rats after unilateral nephrectomy reduced the risk of infection and mortality. Adenine diet given a few days after nephrectomy was effective in preventing the rats from developing rapid malnutrition and death. Unilateral nephrectomy combined with adenine diet for modeling is simple to perform, low in cost and mortality, and deserves to be further promoted or improved upon.

However, no widely accepted method of model preparation exists at present. Further practical studies on the pathogenesis of CKD-MBD will provide new ideas for animal modeling. For instance, proliferation and differentiation of osteoblasts and osteoclasts can be promoted by thyroid hormone (TH); the bone turnover rate is high and the bone remodeling time is short in the hyperthyroid state. Also, previous experiments have shown that the bone turnover rate of rats can be increased by enhancing TH. Therefore, can we supplement TH by thyroxine liquid gavage or other ways based on an adenine diet to cause a high-conversion CKD-MBD animal model? Bone metabolism can be regulated by PTH through different signaling pathways; for example, the Gq/PLC/PKC signaling pathway can be upregulated by increasing PTH, thereby inhibiting bone formation. Therefore, can we upregulate the Gq/PLC/PKC signaling pathway by administering PTH intramuscularly based on the CKD rat model to inhibit bone formation, thus creating a low conversion CKD-MBD animal model with more similar clinical manifestations and more severe vascular calcification? Excessive PTH stimulates increased bone fibroplasia and osteoid formation, and tends to slow down bone mineralization if accompanied by low blood calcium and phosphorus. Then, can we make a mixed CKD-MBD model based on the model of SHPT by decreasing blood calcium and phosphorus in experimental animals through subcutaneous injection of calcitonin, which subsequently leads to insufficient bone mineralization? Finally, osteoarthropathy can be caused by continuous aggregation of β (2)-microglobulin; therefore, can we develop β (2)-microglobulin amyloid osteoarthropathy in experimental animals by local injection of β2-microglobulin at the joints? If the above methods are feasible, they will be more convenient than the existing methods of CKD-MBD modeling, more consistent with clinical characteristics and pathogenesis, and more targeted to create different bone transition types required for experiments. These modeling methods still need to be further studied. Researchers should start from the pathogenesis of CKD-MBD and experimental purposes, find some modeling methods to establish more consistent clinical features and pathogenesis of human CKD-MBD, to provide more new strategies for clinical treatment and disease prevention.

The various methods of modeling CKD-MBD, including surgical, drug and food, radiation, and modified modeling methods, have been established from the pathogenesis and clinical features of CKD-MBD (**Table 1**). Every modeling approach has its own advantages; for example, models with slow disease progression are advantageous because they are more likely to translate to chronic kidney disease in humans, while surgical, radiation, and high-dose adenine models may more often simulate kidney disease after acute kidney injury. However, many animal models of CKD-MBD have the disadvantages of long modeling time, difficult handling, and high mortality (**Table 2**). Therefore, subject selection should be considered by the laboratory workers, including the species, age, weight, and whether the animals have underlying diseases. The operation method, technical requirements, anesthesia dose, time and content of the extraction, and mortality should be controlled by the experimenter for each modeling method.

**Table 1:** Summary of CKD-MBD modeling methods and disease progression-related parameters.

Models	Modeling time	parameter variation	Disease severity	References
5/6 Nx	13 weeks	↑Scr,↑P,↓Ca,↑iPTH, Renal tubular injury, inflammatory interstitial cell infiltration, increased bone resorption, fibrous osteitis and long bone chondromalacia	CKD stage 5	(46, 50–52)
UUO	8 weeks	↑BUN, slightly elevated iPTH, no change in Ca and P, tubulointerstitial fibrosis and tubular atrophy, increased bone resorption	CKD stage 4	(55–58)
Electrocautery	12 weeks	↑P,↑iPTH, no change in Ca, significant depressions in osteoblast number, perimeters, bone formation rates, and mineral apposition rates	CKD stage 4	(59–61)
Adenine diet	4 weeks	↑Scr,↑P,↑iPTH, ↓1,25(OH)_2_D_3_, increased osteoid on the trabecular surface, active osteoblasts, and reduced cancellous bone mineral density	CKD stage 5	(65–69)
High-phosphorus diet	12 weeks	↑Scr,↑P,↓Ca,↑iPTH, renal tubular atrophy, interstitial fibrosis, vascular calcification, and decreased tibial bone volume and mineralization	CKD stage 5	(61)
casein diet	40 weeks	↑Scr,↑BUN,↑P,↓Ca,↑iPTH, SHPT, vascular calcification	CKD stage 5	(75, 76)
Whole body radiation	Within 2 years	↑P,↓Ca, SHPT, Osteochondrosis changes, increased bone remodeling, decreased bone density	CKD stage 5	(81)
Local radiation	12 weeks	Accelerated bone turnover, osteoporosis, fibrous osteitis, resting bone disease and osteochondrosis	CKD stage 5	(82)
5/6 Nx+HPD	12 weeks	↑Scr,↑P,↓Ca,↑iPTH, increased rates of mineral deposition, bone formation, osteoblast circumference and erosion circumference	CKD stage 5	(48, 49, 85, 86)
left nephrectomy+adenine	3 weeks	↑Scr,↑P,↓Ca,↑iPTH, tubular interstitial injury, bone abnormalities	CKD stage 5	(87)
Left nephrectomy+Adriamycin	3 weeks	↑Scr,↑BUN, Renal inflammatory cell infiltration, renal tubular collapse, and low transforming bone lesions	CKD stage 4	(90)
Electrocautery+left nephrectomy	28 weeks	↑Scr,↑P,↓Ca,↑iPTH, Vascular calcification, decreased cortical bone density, decreased bone area and increased osteoclasts	CKD stage 5	(91)
Adenine+phosphorus diet	12 weeks	↑Scr,↑P,↓Ca,↑iPTH, thin femoral cortex, reduced cortical bone mineral density and cortical bone thickness, and reduction in bone volume and trabecular number	CKD stage 5	(92)

5/6 Nx: 5/6 nephrectomy; UUO: unilateral ureteral obstruction; HPD: high-phosphate diet;↑: increased; ↓: decreased; Scr: serum creatinine; P: phosphate; Ca: calcium; iPTH: intact parathyroid hormone; BUN: urea nitrogen; SHPT: secondary hyperparathyroidism; CKD: chronic kidney disease.

**Table 2:** Common animal modeling methods for CKD-MBD and their advantages and disadvantages.

Models	Species	Advantages	shortcomings	Application type	References
5/6 Nx	Rat	stable, widely used, classic and mature	long modeling time, operation difficulty, high mortality, insufficient bone abnormalities and vascular calcification	Chronic renal failure, uremic mixed bone disease, and rapid progression of renal fibrosis	(46, 50–52)
UUO	Rat	mature	Unknown bone metabolic state	Rapid progression of renal fibrosis	(55–58)
Electrocautery	Mouse	reproducible, little bleeding	long modeling time, early Complications, difficult	Uremic mixed bone disease	(59–61)
Adenine diet	Rat	stable, Simple, high repeatability, low mortality, Significant bone disease and vascular calcification	unknown mechanism, weight loss, malnutrition, systemic inflammatory response	Chronic renal failure, highly transformed bone disease	(65–69)
High-phosphorus diet	Mouse	simple, high repeatability, low mortality, similar clinical feature	long modeling time, limited experimental subject, different susceptibility	Lowly transformed bone disease	(61)
casein diet	Rat	simple, no surgery, no drug, simulate disease progression, evaluate diet plan	long modeling time, high Cost	Chronic renal failure, highly transformed bone disease	(75, 76)
Whole body radiation	puppy	definite osteomalacic changes, Similar renal failure progression	high mortality, limited exposure to dogs and radiation sources	Chronic renal failure, uremic mixed bone disease	(81)
Local radiation	Rat	similar clinical feature	long modeling time, the dose and duration of radiation are difficult to control	Chronic renal failure, uremic mixed bone disease	(82)
5/6-Nx+HPD	Rat	similar clinical features, drastic parameter change, pathophysiology examination	SD +Nx: insufficient vascular calcification; SDT +Nx: unknown mechanism, basic disease	Chronic renal failure, highly transformed bone disease	(48, 49, 85, 86)
left nephrectomy+adenine	Rat	significant bone damage and skeletal abnormalities	high risk of surgery	Uremic mixed bone disease	(87)
Left nephrectomy+Adriamycin	Rat	short molding time, drastic parameter change, high repeatability, highly similarity, predictable damage	unknown mechanism, Adriamycin’s batch difference, individual response differences	Lowly transformed bone disease	(90)
electrocautery+left nephrectomy	Mouse	clear mechanism, obvious bone lesions	narrow application range, the complexity of modeling, high mortality	Lowly transformed bone disease	(91)
Adenine+phosphorus diet	Mouse	simple, no surgery, no drug, severe vascular calcification	narrow application range, unknown mechanism, weight loss, malnutrition, systemic inflammatory response	Uremic mixed bone disease	(92)

5/6 Nx: 5/6 nephrectomy; UUO: unilateral ureteral obstruction; HPD: high-phosphate diet.

This review summarizes the current methods for modeling CKD-MBD, their advantages and disadvantages, and scope of application according to the pathogenesis and clinical characteristics of CKD-MBD, combined with the serum biochemical indexes, vascular calcification, and pathological changes of kidney and bone, which provides a more convenient reference for researchers to select, establish, and customize animal models for CKD-MBD research.
